# Effect analysis of percutaneous catheter drainage combined with radiofrequency ablation in the treatment of mixed thyroid nodules

**DOI:** 10.3389/fendo.2025.1644215

**Published:** 2025-08-27

**Authors:** Wencong Sun, Yichen Wang, Chao Ding, Gang Wu, Heng Zhang, Zijie Su, Guoqing Li

**Affiliations:** ^1^ Department of Thyroid Surgery, Henan Provincial People’s Hospital, Zhengzhou University People’s Hospital, Zhengzhou, Henan, China; ^2^ Department of Ultrasound, Henan Provincial People’s Hospital, Zhengzhou University People’s Hospital, Zhengzhou, Henan, China

**Keywords:** minimally invasive treatment, recurrence rate, percutaneous catheter drainage, radiofrequency ablation, cystic thyroid mixed nodules, benign thyroid nodules

## Abstract

**Objective:**

To compare the efficacy of ultrasound-guided percutaneous catheter drainage in the treatment of cystic thyroid mixed nodules.

**Methods:**

A total of 40 patients with cystic mixed thyroid nodules admitted to our hospital were randomly divided into the traditional ablation group (hereinafter referred to as the no-catheter ablation group) and the percutaneous catheter drainage ablation group (20 cases each). The postoperative efficacy of the two groups in the treatment of cystic benign mixed thyroid nodules was compared, including tumor size before and after treatment, treatment duration, pain, hoarseness and recurrence cases.

**Results:**

Compared with the traditional ablation group, the tumor size in the catheter drainage group was significantly reduced, the duration of treatment was shorter, the postoperative pain was lower, the number of hoarseness and tumor recurrence was less (*P*<0.05).

**Conclusion:**

For the treatment of cystic thyroid mixed nodules, percutaneous catheter drainage is more effective than traditional ablation, and the treatment effect is better, the treatment duration is shorter, the postoperative pain is lower, the hoarseness and tumor recurrence cases are less, which is safe and effective. Through clinical study, it is a new and effective treatment method for cystic thyroid mixed nodules, which is worth popularizing.

## 1 Introduction

With the rapid social and economic development in China, the medical industry in our country has also made great progress. In recent years, thyroid nodules have become very common in the normal population, and the detection rate has reached 70%. The incidence of thyroid cancer is also increasing year by year. Compared with thyroid cancer, benign thyroid nodules are more common and about 92.5% of all nodules are benign (1). Radiofrequency ablation for benign thyroid nodules has the advantages of less trauma, smaller scope, more precision and rapid adaptation, which can better reduce the probability of injury of surrounding vital organs, and has been widely used in major diagnosis and treatment centers in China (2). Radiofrequency ablation of benign thyroid nodules can be roughly divided into solid, cystic, solid and cystic mixed nodules according to the nature of nodules. Different from the first three (3–5), after a lot of surgical comparison, it was found that radiofrequency ablation of cystic mixed nodules was not effective, and tumor recurrence and increased complications caused by the re-filling of cystic components of thyroid nodules shortly after surgery were the main problems plaguing thyroid specialists and patients.

## 2 Materials and methods

### 2.1 Study population

A total of 40 patients with thyroid disease admitted to our hospital (from January 1, 2023 to January 31, 2024) with thyroid related lesions mainly cystic thyroid nodules were randomly selected, and were randomly divided into no-catheter ablation group (20 cases) and neck catheter drainage ablation group (20 cases). Randomization was performed using computer-generated random numbers sealed in opaque envelopes. Outcome assessors (ultrasound technicians) were blinded to group allocation. There were 6 males and 34 females aged 15–75 years. The maximum diameter of the nodules is 21-56mm. There was no significant difference in the basic data between the two groups (*P*>0.05). Inclusion criteria: (1) In line with the relevant diagnostic criteria of Expert Consensus on Thermal Ablation of Benign Thyroid Nodules, Microcarcinoma and Cervical Metastatic Lymph Nodes (2018 edition), 40 patients were examined and evaluated by cervical color ultrasound before surgery by doctors with attending and above professional titles in the superficial organ group of our hospital; (2) The largest nodules were mainly cystic mixed nodules. preoperative fine needle aspiration cytological biopsy indicated benign thyroid lesions; (3) Patients who voluntarily signed informed consent for surgery before surgery and were all patients with first onset and receiving treatment; (4) Meet the indications of minimally invasive radiofrequency surgery. Exclusion criteria: (1) Patients with previous thyroid irradiation or related surgical history; (2) Patients who cannot tolerate the treatment related to this study; (3) Patients with severe cardiopulmonary dysfunction and coagulation dysfunction. Informed consent was obtained from all participants. The study was approved by the Ethics Committee of Henan Provincial People’s Hospital (ethics number: MR-41-22-011497). All methods are carried out in accordance with relevant guidelines and regulations.

### 2.2 Surgical procedures

The whole operation was divided into two steps: the first step was percutaneous catheter drainage, and the second step was ultrasound-guided radiofrequency ablation of thyroid nodules.

In the non-catheter ablation group, 20 patients skipped the neck catheter drainage operation and directly underwent ultrasound-guided radiofrequency ablation of thyroid nodules, which was performed by qualified specialists with the title of associate chief physician or above under the guidance of ultrasound. The specific surgical procedures were as follows: The patient was in a supine position with a high neck pad, full exposure to the operative area, routine disinfection and local anesthesia of 2% lidocaine in front of the neck. Under ultrasound guidance, epinephrine injection (1 ml; 1mg) 1 drop of dilute liquid with 100 ml normal saline is injected to form a liquid isolation zone. First, hold a syringe with a 20ml syringe, insert the needle into the cystic component of the mixed nodule, and begin to aspirate until the liquid component can no longer be aspirated. Then, a unipolar/bipolar radiofrequency ablation Electrode (STARmed) [star RF Electrode Fixed 18-07s10F] was inserted into the bottom of the solid component of the thyroid nodule on the affected side, and the solid tumor component and blood supply vessels of the nodule were ablated layer by layer from bottom to top under the condition that the electrode power was adjusted to 65W. The ultrasound image showed as follows: Nodule enhancement was complete and no obvious blood flow signal was observed. After the operation, ultrasound and other imaging equipment were used to determine whether the ablation was complete.

Cervical catheter drainage and ablation group: Another 20 patients underwent cervical catheter drainage and drainage first, as follows: The patient was asked to lie flat, the ultrasonic fixation point was selected as the puncture point, routine skin disinfection was performed, and the 2% lidocaine needle was subjected to local infiltration anesthesia. Under ultrasound guidance, the guiding needle (model 8F) was inserted into the cystic part of the mixed thyroid nodules, and the guide wire was sent, the guide needle was pulled out, the skin was expanded, and the drainage tube was sent along the guide wire. The guide wire was pulled out, and the liquid components and pouch were drained out. The negative pressure drainage device is connected, the drainage tube is fixed, and the operation is finished. The patient was again told to reduce neck and various activities during catheter drainage, keep the neck catheter area dry and change the dressing regularly. After catheter drainage, 20 patients were observed for drainage and the daily cervical fluid drainage was recorded. Observation and record were made every 12 hours. When the continuous drainage volume of the cervical drainage tube was ≤ 2mL for 24 consecutive hours, the drainage area was disinfected, the drainage tube was removed, and then the second step of radiofrequency ablation was performed by the same specialist. The ablation method was roughly the same as that of the non-catheterized ablation group. Ultrasound and other imaging equipment were used to determine whether the ablation was complete.

**Figure 1:**
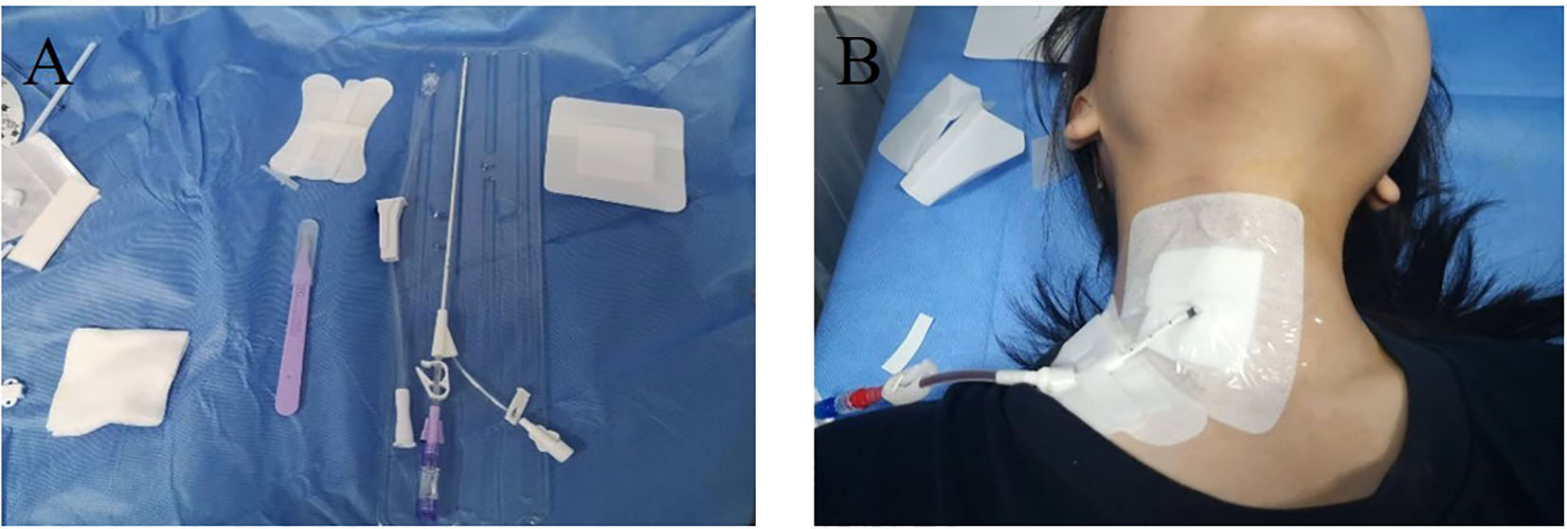
Catheter drainage was performed. **(A)** Shows a single-lumen pig tail tube; **(B)** shows the appearance after neck drainage with drainage tube connected with negative pressure drainage bag.

All procedures were performed by the same operator (with >5 years of thyroid RFA experience) using identical protocols. Pre/postoperative evaluations were conducted by the same surgeon. Photographs of catheter drainage are shown in **Figure 1**.

To minimize perioperative complications, rigorous management prioritized stabilization of vital signs, blood pressure (BP), and blood glucose while eliminating surgical contraindications. Intraoperatively, intravenous parecoxib sodium (40 mg in 100 mL normal saline) was administered for analgesia. Hypertension was controlled through a tiered approach: preoperative oral nifedipine (10 mg, dihydropyridine calcium channel blocker) for systolic BP >140 mmHg, with intraoperative targets maintained at 110–140 mmHg using nitroglycerin micro-infusion (0.5 μg/kg/min via syringe pump) for acute elevations >160 mmHg. Postoperatively, ibuprofen (400 mg orally every 6 hours as needed) addressed fever (>38.0°C), while budesonide nebulization (1 mg/2 mL suspension twice daily) mitigated cough-induced neck tension—a critical measure to prevent bleeding complications exacerbated by respiratory irritation from non-inflammatory exudates. This protocol specifically counters risk in conscious local anesthesia patients, where procedural stress amplifies hypertension and respiratory sequelae.

### 2.3 Index of observation

Related to surgery were observed and compared between the two groups, such as perioperative pain degree, reduction of nodule volume before and after surgery, number of hoarseness cases and number of recurrence cases. The effect of ablation can be determined by the degree of shrinkage of thyroid nodules before and after treatment, nodule volume V=a×b×c×π÷6 (a, b and c are the three largest diametral lines of nodules, respectively), and the reduction rate of ablation lesion volume = (preoperative volume - follow-up volume) ÷ preoperative volume ×100% (6). The degree of pain was assessed by the Visual analogue scale (VAS), with a total score of 0-10, and the higher the score, the more severe the pain. Hoarseness was evaluated according to the number of cases. The advantages and disadvantages of traditional radiofrequency ablation and catheter drainage were evaluated by comparing the difference in the number of recurrence cases.

### 2.4 Statistical analysis

SPSS 25.0 statistical software was used for data analysis. Indicators related to surgery: the number of hoarseness cases, the number of recurrence cases, the duration of surgery, the degree of postoperative pain and the percentage of reduction after nodule ablation were compared. Chi-square test was used to demonstrate whether there were differences in postoperative complications (hoarseness and recurrence) between the two control groups. Whether there were differences in surgical duration, pain degree and percentage of volume reduction after ablation of nodules was demonstrated by t test with two independent samples.

## 3 Results

### 3.1 Clinical characteristics of the two groups before treatment

There were no statistically significant differences in age, gender, nodule size, and nodule location between the two groups (P<0.05). See **Table 1**.

**Table 1:** The clinical data of the two groups were compared.

Grouping	Number of cases	Gender (male/female)	Age ( $\bar{x}\pm s$ )	Tumor location	Tumor size(mm, $\bar{x}\pm s$ )	Number of tumors ( $\bar{x}\pm s$ )
				(left/right/bilateral lobe, number)		
non-catheter ablation group	20	4/16	40.55 ± 12.53	6/9/5	31.35 ± 6.12	2.15 ± 1.14
Cervical catheter drainage and ablation group	20	5/15	40.70 ± 11.01	8/7/5	29.50 ± 4.98	1.80 ± 1.06
*χ^2 /t^*	–	0.14	-0.04	0.56	1.02	1.01
*P*	–	0.71	0.97	0.77	0.32	0.35

### 3.2 Operation-related index

The number of recurrence cases and hoarseness cases in the cervical catheter ablation group was less than that in the non-catheter ablation group, and all hoarseness cases in the two groups recovered effectively within one year after surgery. The duration of operation was less than that of the non-catheter ablation group, and the degree of postoperative pain was lower than that of the non-catheter ablation group. There was no significant difference in the probability of postoperative complications (*P*>0.05), but there were significant differences in the duration of operation and the degree of postoperative pain (*P*<0.05). See **Table 2**.

**Table 2:** Operation-related index.

Grouping	Number of cases	Hoarseness	Recurrence	Operation duration (minute)	Degree of postoperative pain (score)
non-catheter ablation group	20	3	1	24± 8	6± 1
Cervical catheter drainage and ablation group	20	1	0	18± 3	4± 1
*χ^2/t^*	–	2.057		3.14	6.33
*P*	–	0.175		<0.01	<0.01

Chi-square test used for hoarseness/recurrence; independent t-test for operation duration and pain scores.

### 3.3 Tumor related indicators

**Figure 2:**
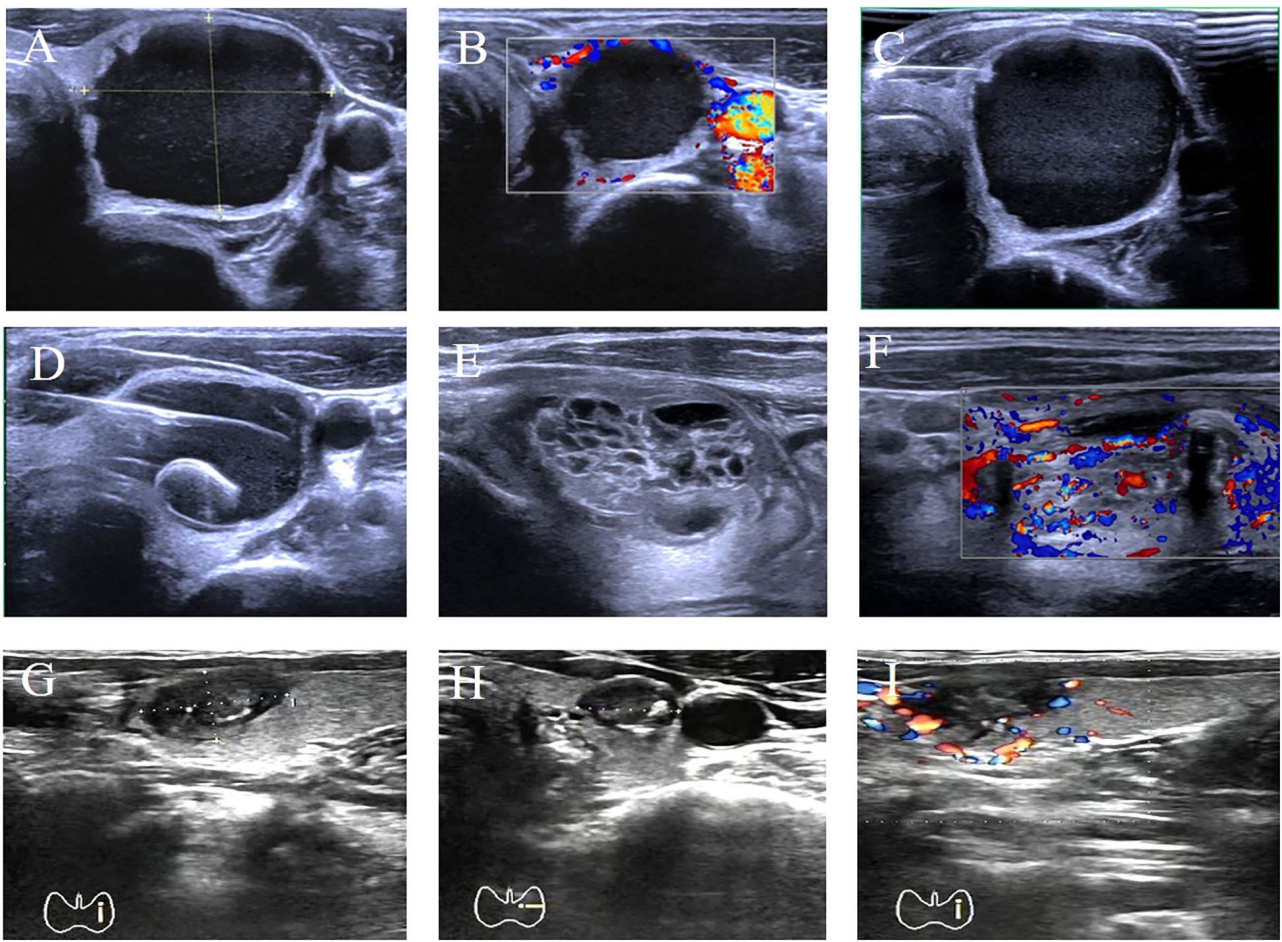
Comparison of preoperative and postoperative results in one patient. **(A)** Transverse plane ultrasound showing a 35×30×23 mm cystic-solid mass (predominantly cystic) in the left thyroid lobe. **(B)** Color Doppler ultrasound demonstrating blood flow signals around the lower pole of the nodule. **(C)** Longitudinal plane ultrasound during fine-needle aspiration biopsy (FNA) of the nodule. **(D)** Transverse plane ultrasound showing placement of a single-lumen pigtail catheter into the nodule. **(E, F)** Longitudinal plane ultrasounds on postoperative day 2 revealing full expansion of the solid component, thickened cyst wall, and appearance of blood flow signals. **(G–I)** Transverse plane ultrasound images at different levels obtained 3 months post-ablation. Ultrasound Imaging Planes: **(A)** Transverse; **(B)** Color Doppler; **(C)** Longitudinal (FNA); **(D)** Transverse (catheter placement); **(E, F)** Longitudinal; **(G–I)** Transverse (post-ablation). Post-ablation nodule size: 13.6×8.6×9 mm. Volume Reduction Rate (VRR): 95.8%.

Using the volume reduction rate (VRR) of the ablation zone as the efficacy evaluation metric for thermal ablation therapy, both groups exhibited significantly decreased tumor volumes at 3 and 6 months postoperatively compared to preoperative baselines (*P*<0.05). The cervical catheter ablation group demonstrated a significantly higher VRR than the non-catheter ablation group, with statistical intergroup difference (*P*<0.05). See **Table 3**. The ultrasound images of one patient before catheter drainage, after catheter drainage, after radiofrequency ablation, and during the follow-up period are shown in **Figure 2**, which shows that the treatment effect is very significant.

**Table 3:** Comparison of thyroid nodule shrinkage between the two groups after treatment ( %, $\bar{x}$ ± *s*).

Grouping	Number of cases	Time after surgery(VRR, %)	
		3 months	6 months
non-catheter ablation group	20	73.6 ± 13.4	85.1 ± 9.1
Cervical catheter drainage and ablation group	20	81.0 ± 10.6	89.8 ± 6.8
*t-value* *p-value*		1.94	1.84
		*P*<0.05	*P*<0.05

*VRR = [(Initial volume - Post-ablation volume)/Initial volume] × 100%.

## 4 Discussion

Radiofrequency ablation has become a promising option for the treatment of benign thyroid nodules. Recent studies have shown that radiofrequency ablation can significantly reduce the volume of thyroid nodules (7, 8). Its advantages are that it is a minimally invasive surgery, which can be performed under local anesthesia in the outpatient department, reducing the risks related to traditional general anesthesia and postoperative recovery time. At the same time, it has no obvious incision in the neck, which meets the urgent needs of patients for neck cosmetology. In a recent meta-analysis, RFA was found to reduce nodule volume by an average of about 50% to 70% in the first year after surgery (9–11). However, the efficacy of radiofrequency ablation alone is not ideal for large cystic and solid thyroid nodules (12).

For large cystic and solid thyroid nodules, the ablation strategies and risks are as follows: 1) intracystic fluid extraction followed by radiofrequency ablation. However, the reduction of intracystic pressure in the solid part of the nodule can easily induce rebleeding of the solid component, which reduces the ablation effect. 2) The solid part was ablated first, then the cystic component was extracted, and finally the residual part was ablated. This procedure is difficult to deal with the thicker cyst wall and is prone to recurrence. 3) ablate the cyst wall first, then aspirate and ablate the solid component. This operation is difficult for cyst wall ablation, because the cyst wall is thin before suction, and the anterior wall of the cyst will produce gas or enhancement after ablation, which will affect the posterior tissue display. 4) Displace the cyst fluid and harden the cyst wall by a sclerosing agent, such as lauromacrogol injection, followed by ablation of the remaining part. The effect of this operation is slightly better than other strategies, but the sclerosing agent can only harden the inner surface of the cyst wall, and can’t avoid re-bleeding on the outer surface of the cyst wall.

After a large number of clinical trials and analyses, the operation of percutaneous catheter drainage is simple and simple. Under ultrasound guidance, a negative pressure drainage tube is placed in the patient’s neck to drain the cystic components of the nodule for 1 to 3 days. The extraction of cystic fluid is accompanied by the gradual decrease of the cystic component and the gradual decrease of the tension, while the relative increase of the solid component and the gradual expansion of the swelling. The coagulation function of the human body is also functioning, the cervical drainage tube no longer has cystic components flowing out, and the solid components bulge completely, occupying all or most of the entire nodule: Further ablation can better observe the swelling and expansion of the solid components of the mixed nodules, and eliminate the interference of the cystic liquid components on the solid components of the nodules, reduce the heat loss of the ablation electrode during surgery, and the ablation treatment has a better effect (13). The ligation of blood vessels in the solid components of nodules can be more refined, thus reducing the probability of postoperative rebleeding and tumor recurrence. In short, prolonged drainage promotes cystic cavity collapse and fibrotic thickening of the cyst wall, converting mixed nodules into predominantly solid lesions. This structural transformation enhances RFA efficacy by reducing heat dissipation into fluid components and enabling precise ablation of expanded solid tissue (8). It should be noted that fine needle aspiration biopsy of thyroid nodules should be performed before catheter drainage, and the benign and malignant nodules should be judged by cytopathology and BRAF gene detection (14–16). Very few malignant thyroid nodules also have cystic-solid imaging features on color Doppler ultrasound. If the malignancy is not ruled out, it may cause tumor spread (17). To avoid false-negative biopsies of mixed nodules, ultrasound-guided biopsies were performed on solid and cystic components, with three or more samples per node sampled, and suspicious areas with blood flow (Doppler) were prioritized (18, 19). Comparison of the results of the two ablation groups in this experiment: There was no statistical difference in postoperative complications (hoarseness and recurrence), which may be related to the small number of cases in the two groups. But the operation duration and pain are significantly lower, and the nodule volume shrinkage rate after treatment is higher, showing a statistical difference. While this study demonstrated significant volume reduction at 6 months, recurrence data beyond 1 year were not collected. Future multi-center studies with extended follow-up (12–24 months) are warranted to validate long-term efficacy.

In short, catheter drainage radiofrequency ablation for the treatment of cystic mixed nodules solves the problem of poor treatment effect and easy recurrence of nodules that has plagued thyroid specialists in our country for many years (20), and the method of catheter drainage for cystic mixed nodules is transformed into the treatment of solid mixed nodules, with good surgical treatment effect. This protocol utilizes standard catheters and RFA devices, making it feasible for resource-limited settings without specialized equipment. At the same time, the postoperative recurrence of patients is reduced, and patient satisfaction is improved, which gives a revelation for the development of minimally invasive thyroid ablation.

## 5 Conclusion

Neck catheter drainage combined with radiofrequency ablation has the advantages of small trauma, quick recovery and good effect in the treatment of mixed thyroid nodules, which is worthy of clinical promotion.

## Data Availability

The raw data supporting the conclusions of this article will be made available by the authors, without undue reservation.
